# The Etiology of Carcinoma

**Published:** 1905-03-11

**Authors:** 


### THE ETIOLOGY OF CARCINOMA.

Dr. Beatson, in a paper read before the Medico-Chirurgical Society of Edinburgh, on March 1st, supported the germinal theory of carcinoma which led him, nine years ago, to suggest the removal of the ovaries in mammary cancer. The writer referred to the recent work of Doyen and that of Ford Robertson and Wade, but held that neither of these observers had fulfilled Koch's postulates in the absence of successful inoculation experiments. Dr. Beatson maintains that removal of the ovaries in patients suffering from mammary cancer has proved of benefit in 36 per cent. of all cases so treated. After oophorectomy the cancer cells degenerate while the stroma increases, the tumour undergoing changes which may lead to its entire disappearance. The changes of the nature of reducing division seen in cancer cells are very similar to those observed by Farmer during the development of normal reproductive tissue. These facts strongly suggest a germinal origin for carcinoma. The almost exclusively cellular character of a cancerous tumour without any attempt at organisation is difficult to reconcile with a microbic origin, as also the absence of signs of irritation when cancer cells find their way into a distant organ. Neither in primary nor in secondary malignant tumours do symptoms arise till the growth has attained sufficient size to act as a foreign body. The delay of patients in applying for advice is related to this, and is to a great extent unavoidable.

The presence of cancer is frequently associated with a change in the body fat which is of a peculiar reddish-yellow colour, and has been ascertained to contain lutein. Whether this change is peculiar to cancer is not known, but the colour of the fat may give rise to the yellow complexion which is characteristic of advanced cases of the disease.

A further analogy between a cancerous tumour and the reproductive organs is to be seen in their relations to the storage of fat by the body, their activity leading to its diminution, while their degeneration or removal favours its accumulation. It may be that the cancer cells perform some physiological part in the elimination of some product of the body or vicariously assist in some function of the reproductive glands. In this connection the nitrifying nodules on the roots of bean plants are of great interest. Should the germinal theory of cancer prove the true solution of the problem there is no reason to despair of successful treatment.

